# The Kang Appliance: A Novel Tooth-Borne Approach to Decompression of Dentigerous Cysts in Pediatric Patients

**DOI:** 10.1155/crid/8858187

**Published:** 2025-07-17

**Authors:** Mary Chapman, Jenny Kang

**Affiliations:** Pediatric Dental Department, UPMC Children's Hospital of Pittsburgh, Pittsburgh, Pennsylvania, USA

## Abstract

Dentigerous cysts are common odontogenic cysts that can cause significant disruption to normal tooth eruption and jaw development in pediatric patients. Traditional marsupialization techniques rely on tissue-borne appliances, which often lead to discomfort and frequent dislodgement in young patients. This case report presents a novel tooth-borne appliance, the Kang Appliance, as an effective alternative for marsupialization.

## 1. Introduction

An 8-year-old male presented with facial swelling and abscessed primary teeth in the lower left quadrant. Radiographic evaluation revealed a large radiolucent lesion suggestive of a dentigerous cyst, causing displacement of the developing permanent premolars and canine. Treatment involved cyst decompression using the newly developed Kang Appliance—a modified distal shoe device designed to maintain an open cystic cavity while providing stability and minimizing patient discomfort. Over a 2-year follow-up, the appliance facilitated controlled cyst resolution and successful eruption of the affected teeth with no recurrence.

Recent literature highlights various techniques for the management of dentigerous cysts, with treatment selection often based on factors such as cyst size, location, patient age, and potential impact on adjacent structures. Enucleation, which involves the complete removal of the cyst lining, remains the treatment of choice when feasible. However, due to the degree of destruction generated by this technique, other more conservative options can be used [1]. Enucleation typically requires the removal of the tooth in question, which is not ideal for developing children.

Marsupialization, a more conservative approach, involves creating a surgical window in the cyst to facilitate continuous drainage and decompression. This technique is particularly beneficial for large cysts or in pediatric patients, as it can reduce cyst size and promote the eruption of associated teeth. In some cases, marsupialization is followed by enucleation to ensure complete removal of the cystic lining [2].

Minimally invasive, two-staged surgical approaches have also been proposed for large cystic lesions of the jaw. This method combines initial decompression to reduce cyst size, followed by definitive enucleation, aiming to minimize surgical morbidity and preserve vital structures [3]. Additionally, endonasal endoscopic techniques have been utilized in specific cases, offering a less invasive option with reduced postoperative discomfort and faster recovery times. This approach is particularly advantageous when the cyst is in proximity to the nasal cavity or maxillary sinus [4].

The choice of treatment should be individualized, considering the specific characteristics of the cyst and the patient's overall condition. A thorough clinical and radiological assessment is essential to determine the most appropriate surgical approach.

## 2. Case Report

This case involves an 8-year-old male patient who presented with abscessed teeth and facial swelling. The patient was initially seen at an express care clinic in Pittsburgh, Pennsylvania, on October 3, 2022, where he was prescribed a 10-day course of amoxicillin due to dental pain and extraoral facial swelling. His medical history was notable for eczema, managed with triamcinolone cream, and he reported allergies to peanuts and eggs, with no known drug allergies (NKDA). His dental history included a complete oral rehabilitation performed on July 9, 2019, by his provider at Children's Hospital of Pittsburgh (CHP), which included multiple stainless-steel crowns on primary teeth, as well as pulpotomy treatment on the primary mandibular first molars.

On clinical evaluation in the CHP dental clinic in 2022, which occurred 1 week after his initial presentation to the express care clinic, the patient reported no pain and exhibited fair oral hygiene with light plaque accumulation. The patient was on Day 6 of a 10-day course of amoxicillin. The extraoral examination revealed no significant findings, while the intraoral examination showed mild swelling in the lower-left vestibule with purulent drainage. A draining fistula noting a soft tissue abscess was noted on Teeth #K, L, and S, and there was evidence of mild gingivitis. The patient's caries risk was classified as severe, and occlusion was assessed as Class I, with a 50% overbite and a 3-mm overjet.

**Figure 1:**
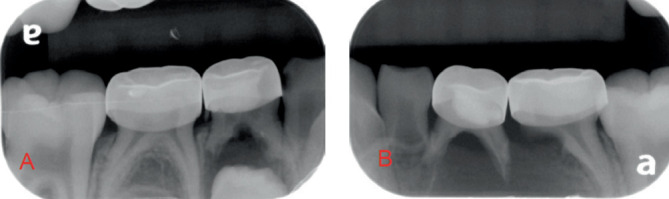
(A) Lower right periapical radiograph showing periapical pathology associated with Tooth #L. (B) Lower left periapical radiograph showing periapical pathology associated with Teeth #K, L, and M.

**Figure 2:**
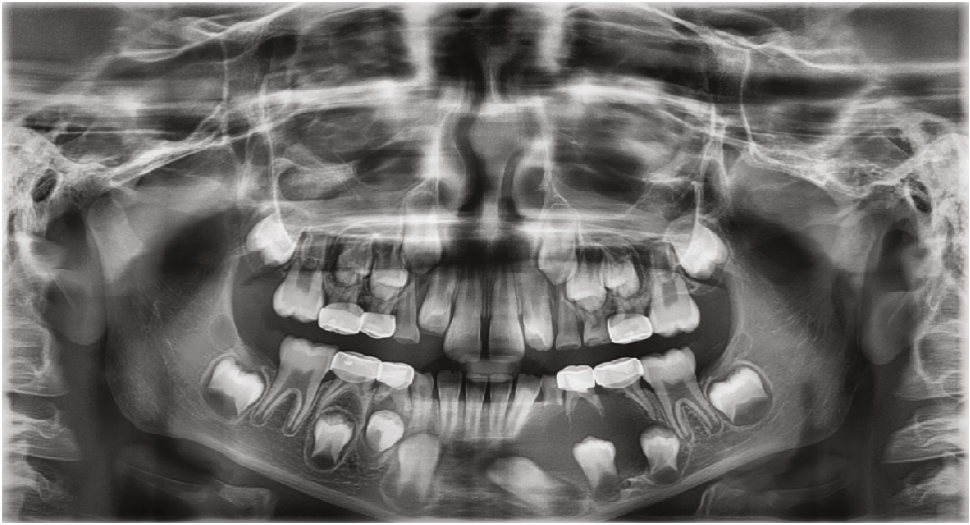
Panoramic radiograph taken at evaluation appointment showing the extent of bony destruction associated with the lower left lesion.

Initial radiographs were taken on October 10, 2022, to assist in formulating the treatment plan. The radiographs (Figures 1 and 2) showed periapical pathology in the furcation of #S and significant radiolucency around the lower left quadrant, including displacement of the succedaneous permanent premolars and canines. Differential diagnoses included a dentigerous cyst, odontogenic keratocyst, and ameloblastoma. Due to the pulpotomy treatment, it is possible that the sequelae are related to inflammation of a failed pulp treatment, such as a buccal bifurcation cyst.

Enucleation of the cyst, marsupialization of the cyst, modified marsupialization and decompression of the cyst, and curettage of the area were considered as possible treatment options. The recommended treatment involved oral conscious sedation using Demerol, Atarax, and nitrous gas, followed by an aspirational biopsy and cyst decompression. Modified marsupialization with a novel decompression technique was chosen for its conservative approach and lower risk of complications compared to enucleation, marsupialization, and the ability to maintain the permanent teeth into adulthood. This approach aligns with previous findings [5, 6] that reported that nearly 62% of dentigerous cyst–associated premolars erupted spontaneously postmarsupialization, especially in young patients with incomplete root development. Additionally, there are fewer post-op complications when opting for marsupialization over enucleation of cysts in children. However, one of the clinical concerns related to marsupialization in children is the chance the drain will get displaced due to the ease of manipulation of the material. Because of this complication associated with tissue-borne appliances, a tooth-borne appliance was developed: the Kang Appliance.

## 3. Treatment

On October 21, 2022, the patient presented to the dental clinic for his sedation and decompression appointment. Informed consent for treatment and sedation with his parent was given and signed. He was given 50 mg of meperidine and 25 mg of hydroxyzine, as well as 40% nitrous oxide for 50 min. The lower left was numbed with 2% lidocaine with 0.017 mg 1:100,000 epinephrine via a mandibular block and 4% articaine with 0.017 mg 1:100,000 epinephrine via infiltration.

**Figure 3:**
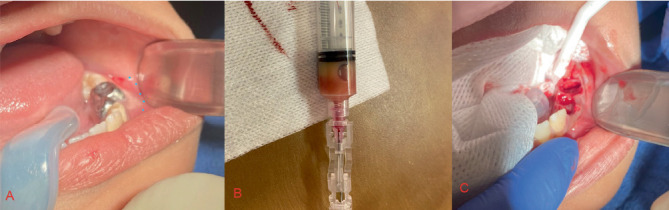
(A) Intraoral swelling in the lower left vestibule associated with Teeth #K and L. The blue dots indicate the extent of the vestibular swelling. (B) Aspirate of the lesion showing straw-colored serosanguinous liquid. (C) Lower left vestibule after extraction of Teeth #K, L, and M.

Once the patient was numbed, an aspirate was taken from the cyst space to confirm the lesion itself was not vascular (Figure 3A). The liquid removed was a straw-colored liquid (Figure 3B), consistent with a cyst and ruling out a vascular malformation. Teeth #K, L, and M in the lower left quadrant were removed, and the cyst lining was identified and opened (Figure 3C).

Marsupialization historically has used one of two approaches at this point. Either the cyst lining was sutured with a long-lasting suture allowing the cyst to involute over time or marsupialization is done by providing a long-lasting Penrose drain that is sutured to the adjacent tissue, allowing the tissue to heal to the outside of the silicone drain but not to the internal surface of the drain. Both of these approaches allow for the cyst to slowly get smaller and close, preventing the cyst from reforming with the quick healing of the oral mucosa.

The problem that was arising in the dental clinic at CHP is that young patients did not like the feeling of the long-lasting sutures or Penrose drains, and subsequently, it would irritate them enough that they would be dislodged, and frequent follow-ups were required to replace the drains. To decrease the burden on the family, we developed a solution with a tooth-borne appliance that would serve the same purpose as the Penrose drain but avoid the complications that arose from a tissue-supported appliance, like dislodgement and irritation.

**Figure 4:**
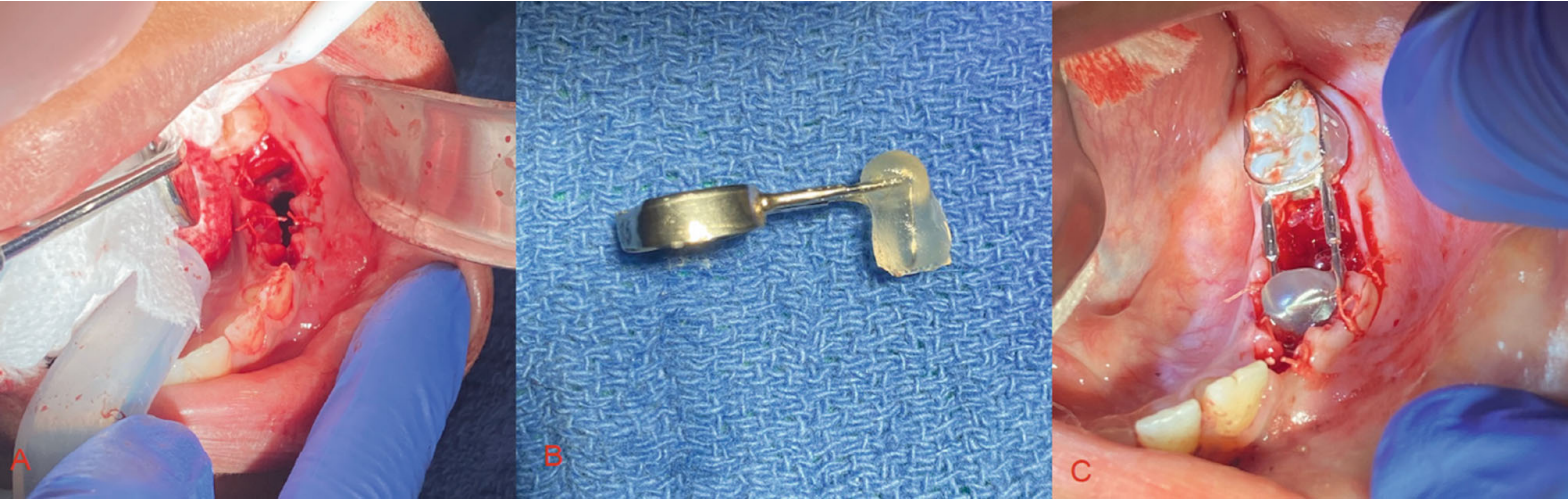
(A) Lower left vestibule after sutures were placed. (B) Kang Appliance made from DeNovo distal shoe surrounded by dental acrylic. (C) Kang Appliance in the mouth after immediate placement on day of extractions.

The appliance was designed and created by a resident and attending utilizing materials that are already common in a pediatric dental clinic. A distal shoe device (DeNovo) was sized so the shoe blade portion infiltrated the center of the cyst (Figure 4B). Acrylic was added to the shoe portion to provide an obturation of the area and allow the mucosa and soft tissue to heal to that point (Figure 4C). A hole was drilled to the middle of the acrylic along the shoe blade to allow for drainage of cystic fluid throughout healing, providing patency without adjustment. These are all normal intraoral pediatric dental materials and were disinfected using CaviCide once the appliance was formed. This design allows for adaptation of the obturator as the cyst heals and guides the premolar into place. The remaining gingival tissue was sutured into place using 6 × 4.0 Vicryl sutures (Figure 4A). The patient was discharged with instructions to use chlorhexidine mouth rinse twice daily for 2 weeks and ibuprofen (5–10 mg/kg q6hr) and acetaminophen (10–15 mg/kg q4hr) for discomfort and maintain a soft diet.

The cystic fluid and a piece of the lining were sent to the University of Pittsburgh Oral Pathology Department for analysis and identification. The pathology report came back as follows: *squamous epithelium with marked inflammation, granulation tissue and fibrosis, consistent with clinical history of dentigerous cyst*.

**Figure 5:**
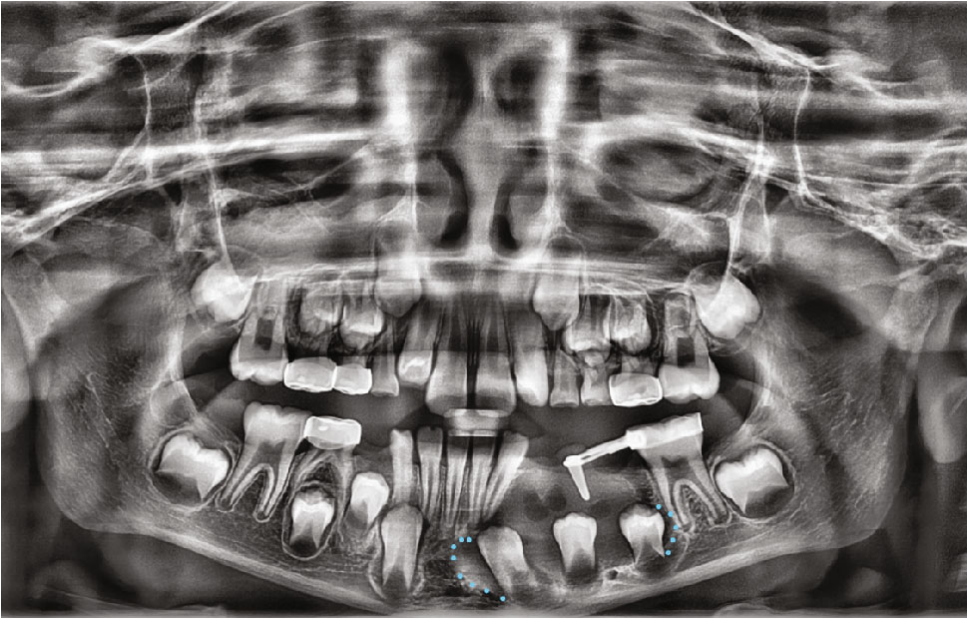
Panoramic radiograph taken 2 weeks after Kang Appliance placement to determine adequate placement. Original lesion size noted by blue dots.

At the 2-week follow-up appointment, an additional panoramic radiograph was taken to confirm appliance placement. Ideal placement was confirmed. The blue outline shows the extensions of the cyst at this time (Figure 5).

**Figure 6:**
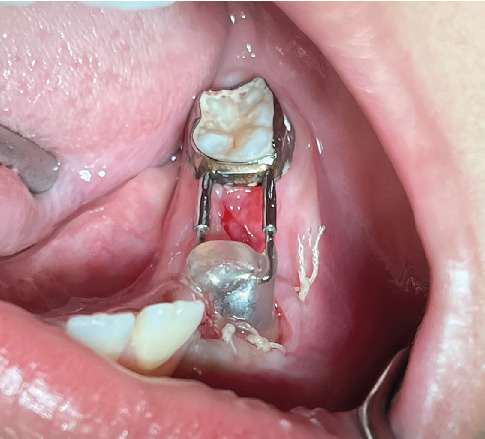
Intraoral view of the Kang Appliance at 2-week follow-up. Note the Vicryl sutures still present and adequate gingival healing. Vestibular swelling is notably absent.

Clinically, the lower left quadrant was healing well, with no signs of infection or pain for the patient (Figure 6).

At the 4-week follow-up, continued cyst healing was noted on a panoramic image. Clinically, the vestibular swelling was markedly improved. Since the lesion was trending in the right direction, our attention shifted to ensuring that we have resolution of the cyst and no abnormal migration or tipping of the 6-year old molar mesially. The device was adjusted so it could have the obturator attached to a lower lingual holding arch to prevent mesial migration of the molar. It was important to design this so the obturator could be removed once it was no longer needed, as seen in the photo. Alginate impressions were taken for fabrication of the new appliance, and models were sent to an outside lab for fabrication.

**Figure 7:**
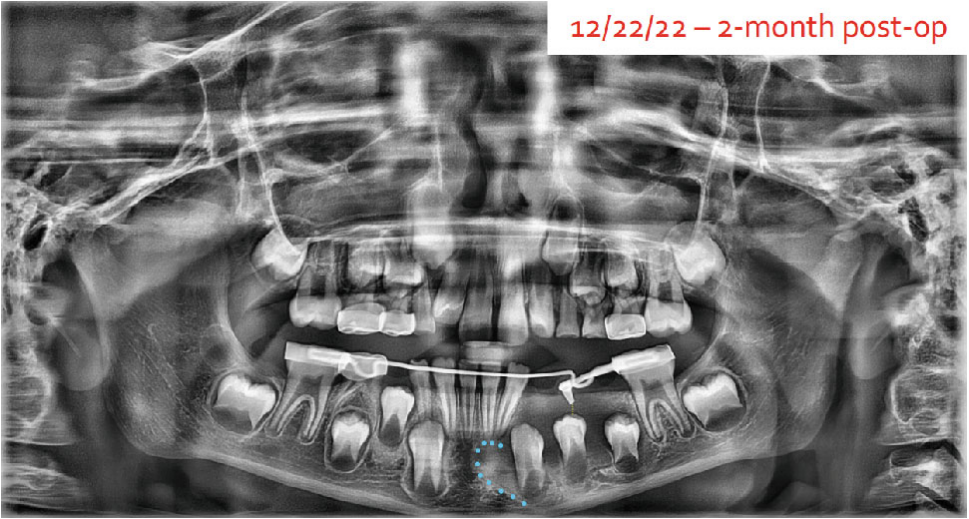
A panoramic radiograph at 2-month follow-up showing Kang Appliance with lower lingual holding arch. Blue dots show original extension of the cystic lesion.

**Figure 8:**
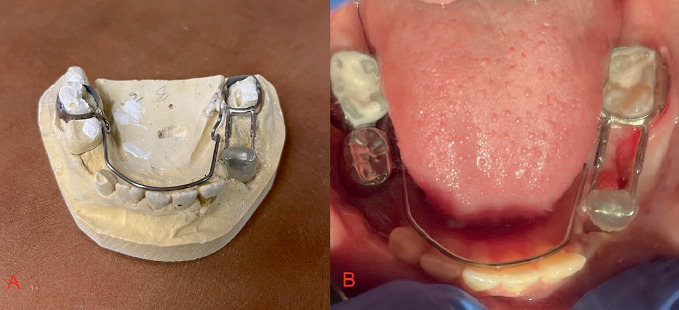
(A) New design of Kang Appliance to incorporate lower lingual holding arch. (B) Mandibular view of Kang Appliance with attached lower lingual holding arch.

At the 8-week follow-up, again the patient was showing great improvement to the cyst resolution on the panoramic radiograph (Figure 7). The new lower lingual holding arch (Figure 8) with the obturator was inserted with the acrylic portion shortened to allow further eruption of the premolar.

**Figure 9:**
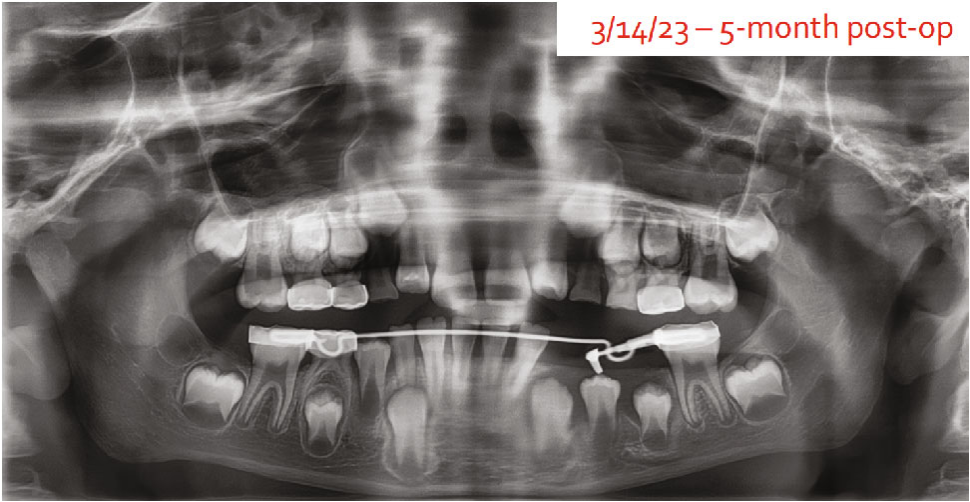
A panoramic radiograph at 5 months post-op shows almost complete resolution of the lesion.

The patient returned for completion of outstanding dental work 5 months after initial cyst decompression, and a panoramic radiograph was taken (Figure 9). It was determined to continue with the obturator in place for another 2 months and then consider removal.

**Figure 10:**
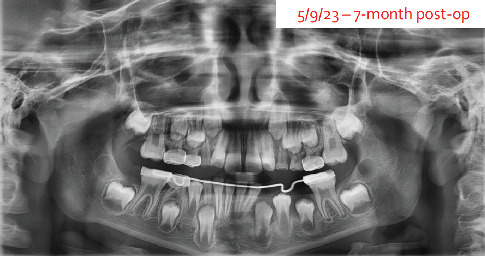
A panoramic radiograph showing Tooth #21 erupting into the oral cavity while the lower lingual holding arch stays in place.

At the next appointment, 7 months post-op, the panoramic radiograph showed that the canine and both premolars are now upright and ready to erupt into place. The distal shoe portion (Kang Appliance) of the lower lingual holding arch was removed, but the lower lingual holding arch remained in place to prevent mesial migration of the 6-year-old molar (Figure 10).

**Figure 11:**
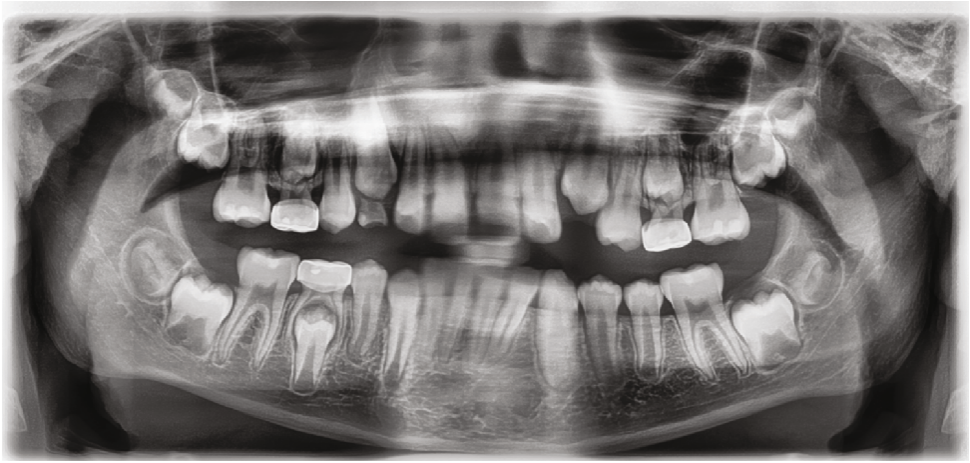
A panoramic radiograph 2 years after operation showing complete bony fill with no residual cyst in the lower left quadrant. The lower lingual holding arch was removed prior to this image. Teeth 20, 21, and 22 are all in ideal position.

Two years after initial presentation, a follow-up panoramic radiograph was taken (Figure 11). Full eruption of both premolars and the canine in the lower left quadrant were noted, along with adequate bony fill of the previous defect, good bone levels, and no indication of any periodontal defects.

## 4. Conclusion

The usage of the Kang Appliance allowed for a tooth-borne cyst obturator for modified marsupialization and decompression. The design allowed for more precise placement and the possibility for fewer complications related to dislodgement. Marsupialization allows for maintenance of permanent teeth associated with dentigerous cysts. The materials used for the Kang Appliance are commonly found in pediatric dental clinics and required very minimal lab work or downtime. The flexibility with design also means that the appliance can be adapted and changed based on the current needs of the patient's dentition.

## Data Availability

The data supporting the findings of this case report (including clinical images and radiographs) are available from the corresponding author upon reasonable request. All patient-identifiable information has been removed to ensure confidentiality.
